# Differential arthropod responses to warming are altering the structure of Arctic communities

**DOI:** 10.1098/rsos.171503

**Published:** 2018-04-18

**Authors:** Amanda M. Koltz, Niels M. Schmidt, Toke T. Høye

**Affiliations:** 1 Department of Biology, Duke University, Box 30338, Durham, NC 27708, USA; 2 Department of Biology, Washington University in St Louis, Box 1137, St Louis, MO 63130, USA; 3 Department of Bioscience, Aarhus University, DK-4000 Roskilde, Denmark; 4 Arctic Research Centre, Aarhus University, DK-8000 Aarhus C, Denmark; 5 Aarhus Institute of Advanced Studies, Aarhus University, DK-8000 Aarhus C, Denmark; 6 Department of Bioscience Kalø, Aarhus University, DK-8410 Rønde, Denmark

**Keywords:** arctic ecology, arthropod, warming, climate change, tundra, community composition

## Abstract

The Arctic is experiencing some of the fastest rates of warming on the planet. Although many studies have documented responses to such warming by individual species, the idiosyncratic nature of these findings has prevented us from extrapolating them to community-level predictions. Here, we leverage the availability of a long-term dataset from Zackenberg, Greenland (593 700 specimens collected between 1996 and 2014), to investigate how climate parameters influence the abundance of different arthropod groups and overall community composition. We find that variation in mean seasonal temperatures, winter duration and winter freeze–thaw events is correlated with taxon-specific and habitat-dependent changes in arthropod abundances. In addition, we find that arthropod communities have exhibited compositional changes consistent with the expected effects of recent shifts towards warmer active seasons and fewer freeze–thaw events in NE Greenland. Changes in community composition are up to five times more extreme in drier than wet habitats, with herbivores and parasitoids generally increasing in abundance, while the opposite is true for surface detritivores. These results suggest that species interactions and food web dynamics are changing in the Arctic, with potential implications for key ecosystem processes such as decomposition, nutrient cycling and primary productivity.

## 1. Background

The rapid warming of the Arctic provides us with a valuable opportunity to learn about the general implications of climate change for the structure and function of biological communities. Temperatures in the Arctic have increased almost twice as fast as the average global increase over the past 100 years [1–4], and its ecosystems are proving to be both sensitive and responsive to such warming [5–9]. Although an abundance of studies has demonstrated the strong effects of climate change on a range of arctic organisms (e.g. [5,9–13]), it is becoming increasingly clear that species responses can be fairly idiosyncratic [14], and that our ability to extrapolate species-level findings to the level of communities may thus be limited [15]. However, a better understanding of community-level responses to climate change is essential, because the structure and composition of communities contribute to how ecosystems function [16].

In spite of the dramatic change in climate that the Arctic has experienced in the recent past, the studies that have explored general responses to this phenomenon have yielded limited evidence of major shifts in community structure. Although warming experiments have shown some site-specific community-wide changes in plant diversity [17,18], a surprising number of long-term experimental [19,20] and observational studies [21–24] have found little-to-no overall change in arctic and alpine plant communities. In particular, compared to some of the compositional changes being observed in low-arctic communities [25–31], ecosystems in the High Arctic still appear to be relatively stable [18,19,22,32]. However, this apparent stability of the High Arctic could simply be an artefact. For example, the typically long lifespans and slow developmental times of many arctic species [33–35], often a product of selection imposed by extremely short growing seasons, pose challenges in detecting changes in population turnover and in identifying the specific environmental stressors responsible for those changes. Similarly, the large inter-annual climatic variability that characterizes extreme environments, such as the Arctic, may obscure temporal trends in population dynamics and complicate the statistical detection of population changes. The latter issue is particularly challenging in arctic datasets because there is some evidence that communities in extreme environments tend to be less dynamic and to exhibit very slow succession rates [36,37]. The use of long-term datasets, which are rare for arctic ecosystems [38], could aid in resolving these issues [39].

Arthropods have long been recognized as a model group for detecting organismal responses to climate change because of their short lifespans and the strong effects that temperature can have on their life histories. As a point of comparison, while tundra plants often live from decades to centuries [33–35,40], the lifespans of most arctic arthropods are much shorter, typically spanning from a few months to several years [41,42]. Thus, while long-lived species such as plants may accumulate subtle changes over the years, arthropods are more likely to show stronger responses to inter- and intra-annual variability in temperature. Nevertheless, there are conflicting predictions on how arthropods will respond to warming in cold environments [43,44]. On the one hand, higher temperatures could reduce population numbers through heat stress [45], desiccation [44], phenological mismatches [46,47], or forced relocation to cooler habitats [48]. On the other hand, warmer temperatures could benefit arthropod species by alleviating thermal cold stress [49] and lengthening the active season, which can facilitate accelerated growth or maturation and potentially result in additional cohorts within the year [41,49,50]. In addition, depending on the ecosystem or habitat-specific responses to warming, increasing temperatures could indirectly facilitate resource acquisition through changes in plant community composition (i.e. food quality) [51], increased rates of primary productivity (i.e. food quantity) (e.g. [52]), or altered availability of substrate and habitat space [53].

The Zackenberg Basic Monitoring Programme at Zackenberg Research Station has consistently monitored climate variables and surface-active arthropod communities in northeastern Greenland since 1996 [54]. This dataset is uniquely suited for the study of community-wide responses to climate change, because it contains information on all trophic levels within the local arthropod community and because the area has undergone extreme summertime warming over recent years (e.g. [47,55,56]). Here, we quantify changes in seasonal temperature patterns at Zackenberg for the period from 1996 to 2014, explore how these changes relate to changes in abundances of different functional groups of surface-active arthropods, and evaluate the extent to which arthropod communities in the three main habitat types (wet fen, mesic heath and arid heath; see [57]) have changed in the recent past. Based on previous short-term studies of arthropod responses to warming (e.g. [44,58,59]), we expected that detritivores would be the most sensitive to a warming climate and that the greatest changes in overall arthropod community composition would occur in those habitats with lower moisture availability (arid and mesic heath).

The climate parameters we consider in our analyses include the mean temperature for the summer in which a sample was collected as well as the mean temperatures for the previous spring, winter, fall and summer. In addition, we consider the duration of winter, because both the temperature and the length of time of exposure to extreme cold can be important determinants of arthropod survival [60–62]. Finally, we also consider the annual number of winter freeze–thaw events, which are another source of temperature-related stress for these animals [49,63].

## 2. Material and methods

### 2.1. Study site

Zackenberg Research Station is located in high-arctic northeast Greenland (74°28' N; 20°34' W). The area is characterized by a continental climate with cold winters and generally dry conditions, and there is continuous permafrost with a maximum active layer depth of 20–100 cm. Mean summertime air temperatures typically vary between 3°C and 7°C [55]. Over the study period of 1996–2014, Zackenberg had a mean annual temperature of −9°C, with the majority of positive average temperatures falling within the summer months of June, July and August [56].

### 2.2. Environmental data

We used hourly data on ambient air temperature measured 200 cm above the soil surface from the Zackenberg climate station (downloaded from data.g-e-m.dk/, accessed 7 October 2015) to calculate conditions for each summer and its preceding spring, winter, fall and summer. We tested for effects of average summer temperature from both the year in which arthropod sampling occurred and one year prior, because the current abundance of arthropods may be partially determined by the conditions during the prior breeding season. While we acknowledge that the most relevant temperature measures for much of the arthropod community are likely to be at the soil surface [64], those data were unfortunately not available for our study plots. However, we note that soil surface temperatures and those at 200 cm above ground were highly correlated over the 19-year study period at the nearby local climate station (Pearson's product-moment correlation: *r*_161 710_ = 0.859), indicating that the metric used here is a reasonable approximation of the temperature experienced by the animals sampled in this study and that the patterns of change over time that we detected at 200 cm are indicative of the changes observed at the surface level. Because air temperature is less likely to vary by microhabitat differences in snow cover or soil moisture, we use this metric in the analyses below. Summer air temperature data were not available from the Zackenberg climate station for 1995 (the year prior to the start of our study), so it was imputed using data from the Climate Research Unit (CRU) TS3.23 Dataset (downloaded from crudata.uea.ac.uk/cru/data/hrg/cru_ts_3.23/, accessed 25 February 2016; see electronic supplementary material, figure S1). Electronic supplementary material, figure S2 and table S2 show the variation in average seasonal temperatures (and winter-related variables; see below) at Zackenberg during the study period.

Operationally, we define winter as the months of November through March, a period with almost exclusively negative temperatures throughout our time-series data. Based on annual seasonal patterns of freeze–thaw cycles, the transition months of April/May and September/October were designated as spring and fall, respectively, and summer was subsequently defined as June, July and August. A more precise measure for the duration of winter was derived using standardized temperature thresholds associated with seasonal freeze–thaw cycles (as in [65]). Specifically, we designated the onset of winter as the last fall date in which there was an hourly temperature measure of 2°C (i.e. start of winter) and the end of the winter as the first spring date at which the temperature reached 2**°**C (i.e. end of winter). The number of freeze–thaw events per winter was calculated as the number of times that the temperature crossed 0°C within that same period. In measuring winter duration, we ignored rare instances where temperatures rose above 2**°**C in the middle of the winter and immediately returned to seasonably cold temperatures [56].

### 2.3. Arthropod data collection

Arthropods were monitored weekly from late May to the end of August from 1996 to 2014 (samples from 2010 were lost in transit from Greenland) in three main habitat types (wet fen, mesic heath and arid heath), which differ in plant community composition, soil moisture and the timing of snow melt [56]. Wet fen habitat is dominated by mosses and grasses, has the highest soil moisture of all habitats and experiences early snow melt; mesic heath is characterized by lichens, *Cassiope tetragona, Dryas* sp. and *Salix arctica*, and late snow melt; arid heath sites are dominated by lichens, *Dryas* sp. and grasses, have relatively low soil moisture and experience early snow melt [57,66]. There were two sampling plots located in mesic heath, two plots in arid heath, and one in wet fen habitat [66]. Although the number of replicates per habitat type is admittedly low, this sampling regime balances the desire to sample all types of local habitats with the effort required to collect long-term data on any single study plot. All plots were located within 600 m of the climate station and were operational during the entire study period, with the exception of one arid heath plot in which monitoring started in 1999. Each plot was 10 × 20 m and contained eight pitfall traps between 1997 and 2006 and four traps in half the original plot (5 × 20 m) during 1996 and 2007–2014. Pitfall traps were one-third filled with saltwater and a few drops of detergent and left out for one week at a time, and all captured specimens were subsequently stored in 75% ethanol. Samples were sorted and counted by technicians from the Department of Bioscience at Aarhus University, Denmark; data are publically available at data.g-e-m.dk/. Because the sampling period was slightly longer in some years than others, we opted to restrict our analyses to samples collected during June–August. Capture numbers within plots were standardized across years by transforming total annual specimen counts to individuals per trap per day. While pitfall traps do not sample the entirety of the arthropod food web (e.g. catches are limited in soil and foliage-dwelling arthropods), they do capture the arthropod community that is active at the soil surface, the organisms of which play an important role in the larger invertebrate tundra food web [67]. Here, we group specimen counts taxonomically by order, except for Collembola and Acari, which were only identified to subclass.

Although weekly counts were available in our plots, we chose instead to analyse sampling data on an annual timescale for two reasons. First, the seasonal dynamics between different arthropod groups are variable, meaning that the arthropod community can have a very different structure from week to week (e.g. [66]). Second, variable weather conditions in a given week, such as solar radiation, can have a large influence on the abundances and types of animals caught in pitfall traps [68,69]. Pooling the total number of arthropods caught over the entire growing season helps to minimize the variance from weekly catches due to this variability. Biases associated with pitfall sampling (see [70]) can also include possible over-representation by highly active and large-bodied animals. Thus, while we acknowledge that these samples are measures of activity densities of arthropods, for simplicity, we refer to the corrected cumulative annual catches (i.e. individuals per trap per day; see above) as ‘abundances' and explicitly assume that among animals frequently caught in pitfall traps, the capture numbers are representative of the proportions of each animal group within the community.

We focused our analysis on the well-represented arthropod groups within the community, which we defined as those groups that were present in at least 85% of our annual samples within a given plot. The animal orders that were sufficiently represented in our sample based on this criterion were Acari (subclass), Araneae, Hemiptera, Hymenoptera, Diptera, Lepidoptera and Collembola (subclass), which represented greater than 99% of the captured arthropods in our plots during the study period. Although organisms like Acari and Collembola are frequently sampled by soil coring, they also comprise an important part of the litter-dwelling community and can be effectively sampled at the soil surface [71]. Some previous studies have found a high degree of spatial synchrony in capture rates and a clearly discernable seasonal dynamic, suggesting that variation in capture rates is driven by environmental factors rather than merely reflecting random processes [66]. Within our samples, there were usually large numbers of mites on bumblebees, so in traps that contained a bumblebee, mites were excluded from analyses so as to not skew the counts of surface-dwelling mites.

In terms of the functional feeding groups of the common arthropods within this ecosystem, Collembola (springtails) and Acari (mites) are generally recognized as detritivores (the composition of surface-dwelling mites at Zackenberg is overwhelmingly dominated by Oribatid mites; see [72]), Araneae (spiders) as predators, Hemiptera (true bugs) and Lepidoptera (moths and butterflies) as herbivores (see [66] for more information on herbivorous species at Zackenberg), Diptera (flies and mosquitoes) as mixed feeders and Hymenoptera as parasitoids (the only Hymenoptera present at this site are wasps that primarily parasitize Lepidoptera) [73–76]. More information on the specific families (and species, where relevant) present at Zackenberg can be found in [66] and [77].

### 2.4. Analyses

Because the climate variables included in our study (i.e. number of winter freeze–thaw events, winter duration and average temperatures during the sampling summer and the previous summer, fall, winter and spring) exhibit high levels of correlation, we avoided statistical artefacts associated with multicollinearity by reducing them to a smaller number of composite predictors via principal components analysis (PCA) in R [78,79]. Variables were centred, scaled and transformed for normality (if needed) prior to PCA. Next, we used linear regression models to explore changes in the underlying components of our climate variables over the 19-year study period. Changes over time for each of the raw climate variables can be seen in electronic supplementary material, figure S2.

We tested for potential habitat-specific effects of our climate variables on arthropod abundances (i.e. cumulative annual catches corrected by trapping days) by fitting separate linear mixed effects models for each taxonomic group. Interactions between habitat type and each of the composite climate parameters derived from PCA were included as fixed predictors. Sampling plot and study year were included as random effects. These mixed models were fitted using the package lmerTest [80,81]. Abundance data for the different taxonomic groups were log transformed in order to conform to the assumptions of linear models [82].

We also performed non-metric multidimensional scaling (NMDS) on the arthropod abundance data to explore how the structure of these communities varied in response to the climate variables (i.e. composite predictors of climate). NMDS is a technique for community analysis that finds an *n-*dimensional ordination of the taxonomic groups that best describes the Bray–Curtis dissimilarity in community composition between plots while minimizing ‘stress’, a measure of badness of fit [83]. Kruskal's stress values of less than 0.2 are generally considered appropriate [84,85]. We ran the NMDS with the Vegan package for R [86] using the metamds function with random starting configurations (maximum of 200 random starts to reach a convergent solution), and we selected the number of ordination axes to keep via visual inspection of a scree plot of stress values [83]. We then tested whether compositional variation at the community level was related to our environmental predictors by fitting the ordination scores to habitat type and the composite climate parameters with Vegan's envfit function [86]. This function is explicitly designed to fit both environmental vectors and factors onto ordination scores [86].

## 3. Results

### 3.1. Environmental trends over the study period

The principal component analysis with varimax rotation revealed three underlying components of climate at Zackenberg. We found a significant change in PC1 over the study period, indicating that active seasons (summers and falls) became progressively warmer and that winters in later years had fewer freeze–thaw events (*F*_1,16 _= 16.98, *p < *0.001, *r*^2^ = 0.51). This finding is consistent with previous reports of summertime warming at Zackenberg over recent years (e.g. [47,55,56]). There was no evidence of significant changes in the temperature of the non-active season (winters and springs; PC2; *p *= 0.43) or in winter duration (PC3; *p *= 0.44) over the study period. The relative contributions of each climate variable to the different components derived from the PCA (i.e. ‘warmer active seasons and fewer freeze–thaw events’, ‘warmer non-active seasons' and ‘longer winters') are presented in table 1.

**Table 1.** Climate variable loadings from Zackenberg, Greenland (1996–2014) onto principal components. Main contributors for each principal component (greater than 0.50) are highlighted in boldface type. Temperatures include the average seasonal temperature for each summer of sampling and for each season over the previous year. Average temperature of the previous summer was log-transformed prior to PCA.

climate variable	warmer active seasons & fewer winter freeze–thaw events (PC1)	warmer non-active seasons (PC2)	longer winters (PC3)
average summer temperature	**0**.**87**	0.1	0.26
average temperature of previous summer	**0**.**76**	0.47	−0.03
winter freeze–thaw events	−**0**.**69**	0.17	−0.04
average temperature previous fall	**0**.**67**	0	−0.43
average spring temperature	−0.06	**0**.**81**	−0.35
average winter temperature	0.1	**0**.**78**	0.38
winter duration	0.06	−0.02	**0**.**91**
eigenvalue	2.27	1.52	1.35
% variation	0.32	0.22	0.19
cumulative % variation	0.32	0.54	0.73

### 3.2. Links between arthropod abundances and environmental predictors

In total, our analyses included 593 788 arthropods captured during June–August from 1996 to 2014. An average of 9185 (arthropod) specimens were caught per year in the single wet fen plot, 10 308 specimens in the two mesic heath plots, and 13 495 specimens in the two arid heath plots. While pitfall traps may lead, in some instances, to biased sampling with overrepresentation of large-bodied and/or more active arthropods, we note that smaller-bodied arthropods and groups that are often under-sampled with this method (like detritivores) were nevertheless notably well represented in our traps (electronic supplementary material, figure S3). Electronic supplementary material, figure S3 also shows the variation in annual abundances of each taxonomic group during the study period.

We found that habitat type is an important predictor of the abundance of most taxonomic groups. In particular, predators (Araneae) and flies (Diptera) were more abundant in the wet fen than in the heath sites (table 2), whereas the opposite was true for herbivores (Hemiptera and Lepidoptera). There were also fewer Acari in the mesic heath than in the arid heath sites (table 2).

**Table 2.** Mixed effects model results of summertime abundances of the most common arthropods at Zackenberg, as predicted by habitat type and the three principal components summarized in table 1. Higher values of PC1 are indicative of warmer active seasons and fewer winter freeze–thaw events; higher values of PC2 represent warmer non-active seasons, and higher values of PC3 are indicative of longer winters. Interactions between habitat type and each of the composite environmental variables were included in all models as fixed effects, whereas Plot and Year were included as random effects. Arid heath is the reference category for habitat type. Individual models were simplified by removing non-significant predictors one by one. Results shown here are from the most parsimonious models. *p < 0.05, **p < 0.001, ***p < 0.0001.

fixed effects terms	coeff.	s.e.	d.f.	*t*	*p*-value
*Acari*					
intercept	1.220	0.122	15.25	10.007	<0.001***
habitat type (reference category: arid heath)					
mesic heath	−0.499	0.110	4.23	−4.543	0.009**
wet fen	0.326	0.134	4.13	2.441	0.069
PC1	−0.231	0.120	30.95	−1.935	0.062
PC2	—	—	—	—	n.s.
PC3	−0.233	0.115	26.70	−2.029	0.053
habitat type × PC1					
mesic heath	0.201	0.095	66.49	2.110	0.039*
wet fen	0.206	0.114	65.97	1.816	0.074
habitat type × PC2					
mesic heath	—	—	—	—	n.s.
wet fen	—	—	—	—	n.s.
habitat type × PC3					
mesic heath	0.116	0.089	64.86	1.302	0.198
wet fen	0.274	0.108	64.81	2.528	0.014*
*Araneae*					
intercept	0.636	0.065	8.21	9.779	<0.001***
habitat type (reference category: arid heath)					
mesic heath	0.149	0.078	4.66	1.896	0.121
wet fen	0.364	0.096	4.61	3.804	0.015*
PC1	—	—	—	—	n.s.
PC2	−0.085	0.047	33.24	−1.813	0.079
PC3	0.052	0.047	33.48	1.104	0.277
habitat type × PC1					
mesic heath	—	—	—	—	n.s.
wet fen	—	—	—	—	n.s.
habitat type × PC2					
mesic heath	0.115	0.046	64.91	2.514	0.014*
wet fen	−0.023	0.056	64.86	−0.410	0.684
habitat type × PC3					
mesic heath	0.019	0.046	64.97	0.407	0.685
wet fen	−0.123	0.056	64.90	−2.208	0.031*
*Collembola*					
intercept	1.268	0.222	6.17	5.721	0.001**
habitat type (reference category: arid heath)					
mesic heath	0.147	0.295	4.90	0.498	0.640
wet fen	−0.391	0.361	4.89	−1.084	0.329
PC1	−0.181	0.084	18.07	−2.151	0.045*
PC2	−0.220	0.083	17.44	−2.640	0.017*
PC3	−0.295	0.094	27.79	−3.138	0.004**
habitat type × PC1					
mesic heath	—	—	—	—	n.s.
wet fen	—	—	—	—	n.s.
habitat type × PC2					
mesic heath	—	—	—	—	n.s.
wet fen	—	—	—	—	n.s.
habitat type × PC3					
mesic heath	0.070	0.078	64.99	0.891	0.376
wet fen	0.239	0.095	64.95	2.509	0.015*
*Diptera*					
intercept	1.037	0.091	33.65	11.383	<0.001***
habitat type (reference category: arid heath)					
mesic heath	0.070	0.084	69.62	0.830	0.410
wet fen	0.877	0.102	69.51	8.575	<0.001***
PC1	—	—	—	—	n.s.
PC2	—	—	—	—	n.s.
PC3	—	—	—	—	n.s.
habitat type × PC1					
mesic heath	—	—	—	—	n.s.
wet fen	—	—	—	—	n.s.
habitat type × PC2					
mesic heath	—	—	—	—	n.s.
wet fen	—	—	—	—	n.s.
habitat type × PC3					
mesic heath	—	—	—	—	n.s.
wet fen	—	—	—	—	n.s.
*Hemiptera*					
intercept	0.212	0.039	8.28	5.372	0.001***
habitat type (reference category: arid heath)					
mesic heath	0.081	0.048	5.10	1.701	0.148
wet fen	−0.148	0.058	4.97	−2.555	0.051
PC1	0.122	0.039	57.34	3.159	0.003**
PC2	—	—	—	—	n.s.
PC3	0.063	0.036	47.07	1.779	0.082
habitat type × PC1					
mesic heath	−0.100	0.044	67.36	−2.257	0.027*
wet fen	−0.088	0.053	66.60	−1.674	0.099
habitat type × PC2					
mesic heath	—	—	—	—	n.s.
wet fen	—	—	—	—	n.s.
habitat type × PC3					
mesic heath	−0.140	0.041	65.12	−3.375	0.001**
wet fen	−0.093	0.051	65.01	−1.831	0.072
*Hymenoptera*					
intercept	0.162	0.039	6.36	4.140	0.005**
habitat type (reference category: arid heath)					
mesic heath	0.101	0.052	5.09	1.935	0.110
wet fen	0.027	0.064	5.04	0.421	0.691
PC1	0.016	0.025	55.61	0.632	0.530
PC2	0.015	0.023	44.87	0.664	0.510
PC3	—	—	—	—	n.s.
habitat type × PC1					
mesic heath	0.090	0.028	66.84	3.216	0.002**
wet fen	0.003	0.034	66.32	0.099	0.921
habitat type × PC2					
mesic heath	0.069	0.026	65.30	2.638	0.010*
wet fen	−0.049	0.032	65.23	−1.542	0.128
habitat type × PC3					
mesic heath	—	—	—	—	n.s.
wet fen	—	—	—	—	n.s.
*Lepidoptera*					
intercept	0.152	0.020	27.48	7.492	<0.001***
habitat type (reference category: arid heath)					
mesic heath	−0.010	0.016	68.84	−0.644	0.522
wet fen	−0.100	0.020	68.77	−5.107	<0.001***
PC1	—	—	—	—	n.s.
PC2	—	—	—	—	n.s.
PC3	—	—	—	—	n.s.
habitat type × PC1					
mesic heath	—	—	—	—	n.s.
wet fen	—	—	—	—	n.s.
habitat type × PC2					
mesic heath	—	—	—	—	n.s.
wet fen	—	—	—	—	n.s.
habitat type × PC3					
mesic heath	—	—	—	—	n.s.
wet fen	—	—	—	—	n.s.

**Figure 1.**
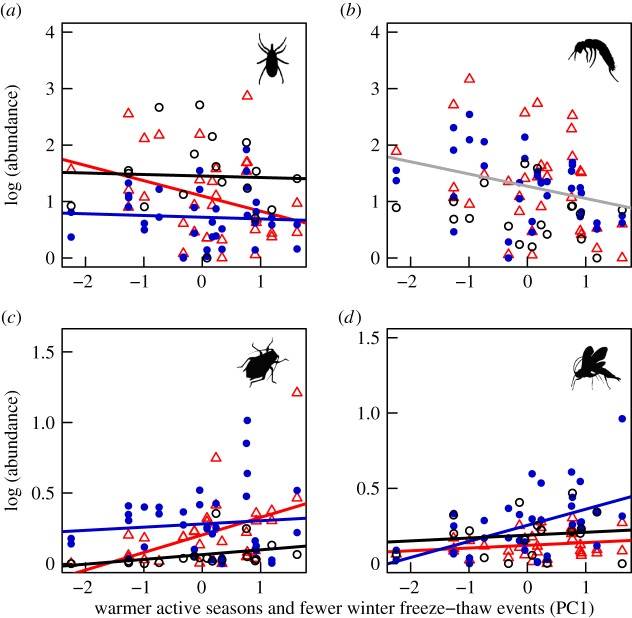
Summertime abundances of (*a*) Acari, (*b*) Collembola, (*c*) Hemiptera and (*d*) Hymenoptera as predicted by the first principal component from the PCA (warmer active seasons and fewer winter freeze–thaw events). The habitat types are wet fen (black circles), mesic heath (blue filled circles) and arid heath (red triangles). Grey trend lines show significant relationships between average daily animal abundances and PC1, whereas the coloured trend lines signify a significant interaction between PC1 and habitat type. Results from these mixed effects models and those from the other arthropod groups can be found in table 2.

**Figure 2.**
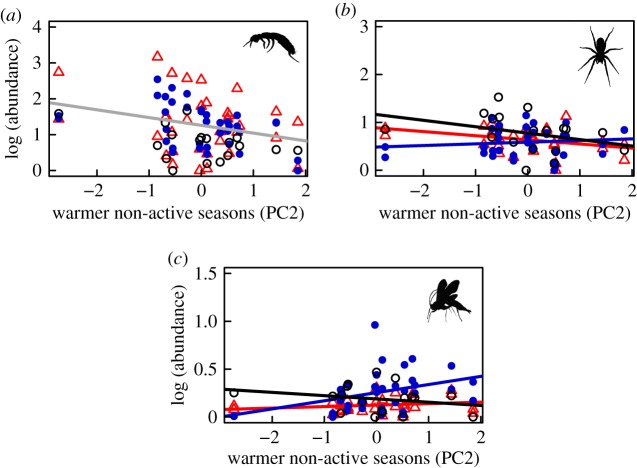
Summertime abundances of (*a*) Collembola, (*b*) Araneae and (c) Hymenoptera in relation to variability in PC2 (warmer non-active seasons). Habitat types are denoted by colour and symbol type: wet fen (black circles), mesic heath (blue filled circles) and arid heath (red triangles). Solid coloured lines signify a significant interaction between PC2 and habitat type in relation to abundance. Collembola abundances were negatively related to variability in PC2, independent of habitat type (depicted by single grey trend line). Results from these mixed effects models and those from the other arthropod groups can be found in table 2.

**Figure 3.**
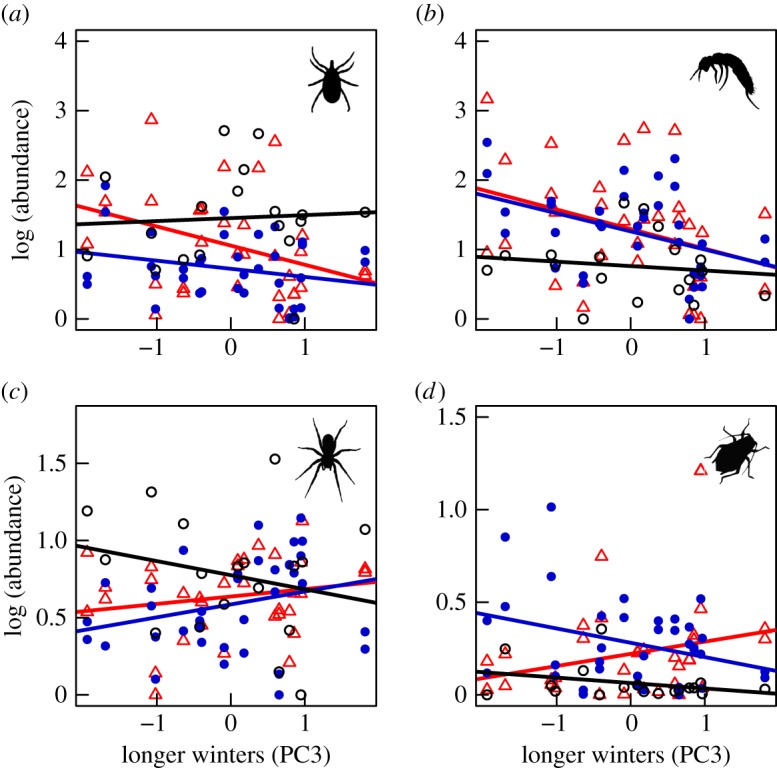
Summertime abundances of (*a*) Acari, (*b*) Collembola, (*c*) Araneae and (*d*) Hemiptera in relation to variability in PC3 (longer winters). The different habitat types are wet fen (black circles), mesic heath (blue filled circles) and arid heath (red triangles). Solid coloured lines signify a significant interaction between habitat type and longer winters. Results from these mixed effects models and those from the other arthropod groups can be found in table 2.

We also found that the magnitude and direction of climate-related responses varied among taxa and were highly dependent on habitat type (table 2). For example, when active seasons were warmer and winters had fewer freeze–thaw events (higher PC1 scores), surface detritivores were present in lower abundances (Acari only in arid heath, Collembola in all habitats: figure 1*a*,*b*). By contrast, Hemiptera and parasitoid Hymenoptera were found to be more abundant in relation to higher PC1 scores (figure 1*c*,*d*), particularly in arid heath (Hemiptera) and mesic heath (Hymenoptera) habitats. Similarly, warmer non-active seasons (higher PC2 scores) were found to correlate with lower Collembola abundance (figure 2*a*) and lower spider abundance in wet fen and arid heath (figure 2*b*) but higher abundance of parasitic Hymenoptera in mesic heath (figure 2*c*). Longer winters (higher PC3 scores) were correlated with lower abundances of surface detritivores in both heath habitats (figure 3*a*,*b*) and with habitat-specific changes in spiders (figure 3*c*) and Hemiptera (figure 3*d*). No climate effects were detected on Diptera or Lepidoptera, but the abundances of both of these groups varied significantly between habitats (table 2). Overall, these results indicate that certain functional groups (e.g. detritivores such as Collembola and Acari) may be more sensitive to climatic variation than others.

### 3.3. Links between variability in community structure and environmental predictors

**Figure 4.**
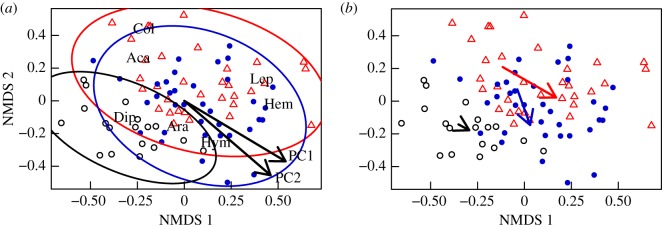
NMDS ordination of annual arthropod communities from Zackenberg, Greenland between 1996 and 2014 in wet fen (black circles), mesic heath (blue filled circles) and arid heath (red triangles) habitats. Each point represents the community composition from a single plot within a given sampling year. (*a*) PC1, indicative of warmer active seasons and fewer winter freeze–thaw events, and PC2, indicative of warmer non-active seasons, are overlaid as correlation vectors, whereby the arrows show the direction of the gradient, and the length of the arrows are proportional to the correlations between the climatic variables and the ordination (PC1: *p = *0.004; *r*^2^* *=* *0.13; PC2: *p *= 0.003; *r*^2^* *=* *0.12). Abbreviations in black denote the centroids of each of the analysed arthropod groups (Col, Collembola; Aca, Acari; Dip, Diptera; Lep, Lepidoptera; Ara, Araneae; Hem, Hemiptera and Hym, Hymenoptera); habitat-specific communities are delimited by 95% confidence ellipses. (*b*) Arrows indicate the change in average NMDS scores for communities in each habitat type from the first third (1996–2001) to the last third of the study period (2009–2014).

Non-metric multidimensional ordination (NMDS) of arthropod community composition resulted in a two-axis solution and a Kruskal's stress value of 0.195 (figure 4*a*). This ordination analysis confirms that arthropod community composition differs according to habitat type (*p *< 0.001; *r*^2^ = 0.29), with communities within mesic and arid heath habitats being more similar to one another than to those within wet fen habitat.

NMDS also indicates that community composition within these high-arctic communities during our 19-year study period was significantly related to variation in PC1 (warmer active seasons and fewer freeze–thaw events; *p = *0.004; *r*^2^ = 0.13) and PC2 (warmer non-active seasons; *p = *0.003; *r*^2^ = 0.12). The nature and direction of these associations are depicted graphically in figure 4*a* through correlation vectors [86]. Owing to the observed shift towards warmer active seasons and fewer freeze–thaw events over our study period (see section on Environmental trends above), we can infer that those communities present in years with cooler active seasons and more freeze–thaw events (lower values of PC1) were communities that occurred in the beginning of the study period while those present in years with warmer active seasons and fewer freeze–thaw events (higher values of PC1) occurred later. Although variability in arthropod community composition was also significantly linked to warmer non-active seasons, we note that there is currently little evidence for significant changes in non-active season temperatures at Zackenberg in the recent past.

Overall, our ordination analysis paints a picture that is consistent with our analyses on individual arthropod groups: as active seasons have become warmer and winters have brought about fewer freeze–thaw events, Zackenberg's arthropod communities have seen an increase in the number of herbivores (Hemiptera) and parasitoids (Hymenoptera) and a decrease in detritivores (Collembola and Acari). NMDS also indicates that temporal shifts in community composition have been more pronounced in arid and mesic heath habitats than in the wet fen. For example, between the first third (1996–2001) and the last third (2008–2014) of the study period, changes in the average NMDS scores of arid and mesic heath communities were respectively about five and three times larger than those from the wet fen community (figure 4*b*), an observation which is robust to comparisons between different 6-year time windows. Considering our findings on taxon-specific responses to climate variation, it seems likely that the slight differences in community-level changes between the arid and mesic heath (figure 4*b*) result from differential habitat-specific responses by Hemiptera and Hymenoptera to warmer active seasons and fewer freeze–thaw events (table 2 and figure 1*c*,*d*). Specifically, the finding that abundances of Hemiptera increased more in the arid heath than in the mesic heath (figure 1*c*), whereas those of Hymenoptera showed the opposite pattern (figure 1*d*) is consistent with the way in which these groups are expected to respond to increasing temperatures and fewer freeze–thaw events (changes in PC1 and PC2; figure 4*a*,*b*).

## 4. Discussion

We have shown that over a 19-year period in the recent past, the Zackenberg area of NE Greenland has experienced significant warming of active seasons and progressively fewer winter freeze–thaw events. In addition, our analyses demonstrate that these changes in climate are associated with major axes of change in the composition of local arthropod communities and are correlated with significant changes in community structure over time. Specifically, abundances of surface detritivores are declining with the increasingly warmer active seasons and reduced number of freeze–thaw events, whereas abundances of parasitoids and certain herbivores are increasing.

Although the long-term dataset that our study is based upon was not originally designed to explore differences among habitat types (i.e. replication within habitat types is low), our analyses were nevertheless still able to uncover that taxon-specific and community-level responses to changing climatic conditions at Zackenberg can be habitat-specific and are likely to vary across small spatial scales (e.g. such as the short distances between our research plots). For example, we observed much larger changes in arthropod community composition in association with active season warming and winter freeze–thaw events in the arid and mesic heath sites than in the wet fen habitat. These results are consistent with earlier claims that climatic changes can elicit differential responses in habitats that vary in moisture availability [59,87,88], as soil moisture has direct effects on desiccation-susceptible arthropods and can also have indirect effects via changes in plant palatability [89]. In addition, water availability plays an important role in the overwintering strategies of many arctic arthropods [90]. The potential for habitat-specific responses could also be driven by differences in temperature and other microhabitat conditions at the soil surface, which can vary depending on vegetation type and snow cover (e.g. [27,88,91–93]). For example, one possible reason why mesic heath communities exhibit slightly different changes than those we observe in arid heath or wet fen (figure 4*b*) is that mesic heath sites experience later snow melt than the other habitats [66] and therefore may select for different phenologies [92], temperature-associated responses or species interactions. The latter can be seen in the way in which warmer active seasons in mesic heath habitats are associated with higher abundances of parasitoid wasps but not of their Lepidoptera prey. This difference suggests that parasitoid pressure on Lepidoptera may be increasing over time in mesic heath. Similarly, the higher abundances of Hemiptera in arid heath but no change in potential spider predators indicate that predator pressure could be declining in those areas. While arthropod communities that inhabit heath versus fen sites are compositionally distinct from one another (figure 4*a*), it is possible that movement by individuals across the mosaic landscape could somewhat buffer communities from the altered species interactions caused by changing abundances. However, our results suggest that overall, differences in habitat-specific responses by arthropod groups to climatic changes may be altering the strength of some predator–prey interactions, particularly in heath habitats.

Notably, although arthropod communities in drier habitats appear to be more responsive to climate change than those in wet habitats (figure 4*b*), the opposite appears to be the case for plants [24,26,57]. This difference in habitat-specific responses by the plant and arthropod communities suggests that increasing temperatures could magnify the existing spatial heterogeneity across the landscape. For example, while we might have expected herbivores (i.e. Hemiptera) to respond positively to the increase in plant productivity in wet fen habitat that occurred during the course of our study [94,95], we found instead that the herbivore abundance remained largely stable there. Assuming that rates of herbivory scale with the abundance of herbivores, this finding suggests a proportional decrease in invertebrate herbivory in wetter habitats. Conversely, although plant communities in drier heath habitats have been relatively resistant to warming [24,26], herbivore abundances have increased in arid heath (figures 1*c*, 4*a*), potentially leading to higher rates of herbivory in those areas. This mismatch between plants and herbivores supports the hypothesis that northern herbivores are more limited by abiotic conditions than by resource availability [96]. A number of studies have shown how vertebrate herbivores can influence how warming affects arctic plant communities with important consequences for carbon exchange [97–103]. Less attention has been paid to changes in the lower baseline herbivory rates by arthropods, but our findings are consistent with results from previous modelling efforts [104] and observations on *Betula nana* and *B. glandulosa* [105] that suggest that impacts on vegetation by non-outbreak herbivores will increase with the predicted increases in temperature. Moreover, we may have even underestimated the herbivore response to warming in all habitats, because our trapping method was not focused on sampling the foliage community, which has a higher proportion of herbivores than the surface-dwelling community [67]. The increases in herbivore abundances that we observed in the arid heath are probably reducing the small, albeit increasing amount of carbon that is annually fixed and retained in this arctic ecosystem ([94], but see [106]).

Our results are also consistent with prior claims that above-ground and soil-dwelling arthropods might respond differently to warming and that while warming may benefit herbivores (e.g. [107,108]), it will be detrimental to detritivores (e.g. [44,58,59]). We found that detritivores in this system, particularly Collembola, are extremely sensitive to temperature-related changes in their environment. A number of previous studies on experimental climate warming have shown that Collembola respond negatively to increasing temperatures but that these responses are more likely due to changes in moisture associated with warming than the warming itself ([44] and references therein). Detritivores with more heavily sclerotized forms (e.g. mites such as Oribatida and Mesostigmata) are more resistant to desiccation and warming [44,109], which may explain why we only observed a decline in Acari in the arid heath but not the mesic heath or wet fen (figure 1*a*). The ecosystem implications of having relatively fewer surface-active detritivores in these arthropod communities are unclear (but see [67]). Soil animals are commonly found to enhance decomposition [110–113] and nutrient cycling [114–116] through their consumption of litter and interactions with the microbial community. In this case, lower detritivore densities in the litter may translate to slower decomposition on the tundra, which could slow the rate at which the large amounts of stored carbon are released from permafrost soils. At the same time, if lower abundances of detritivores result in fewer plant-available nutrients, like nitrogen, there could be limitations imposed on primary productivity [114] which would slow the rate at which carbon is fixed. However, predicting future impacts of warming on detritivore-mediated processes are inherently difficult, because they largely depend on the specific composition of local detritivore and microbial communities [117]. Furthermore, while our results demonstrate the negative effects of long-term warming on both groups of surface-active detritivores, these findings may not always extend to communities further below ground (e.g. [118,119]), where differences between Collembola and mite responses to experimental warming have been recorded (e.g. [59,120–122]). Overall, the uncertainty surrounding the sensitivity of arctic detritivores and their influence over ecosystem processes under different environmental conditions highlights the demand for more studies focused on the functional role of these animals [123].

Even though summer and early fall are the active season for most arctic arthropods, we found that conditions during the preceding winter were also related to arthropod abundances and community structure. In particular, abundances of all except two arthropod groups (Diptera and Lepidoptera) were associated with either the length of winter (PC3) or the mean temperature during the non-active season (PC2). These patterns are consistent with earlier claims that cold-adapted polar arthropods are sensitive to winter conditions, even during periods of diapause [49,65,124–128]. While we did not observe long-term changes in mean temperature in the non-active season at Zackenberg, climate projections for Greenland suggest both winter and springtime will eventually be warmer [129]. Thus, our results indicate that predictions regarding the potential type and magnitude of future changes in northern arthropod communities will require accounting for warming in both the active and non-active seasons.

In conclusion, we have shown that long-term warming has differentially affected arthropod groups in high-arctic Greenland, leading to major changes in the relative abundances of different trophic groups within the arthropod community. These changes were especially pronounced in the dry habitats, suggesting that the strength of organismal responses to warming is likely to be habitat-specific and linked to moisture availability. Potential avenues to expand on these findings include increasing taxonomic resolution and replication within habitat types, as well as explicitly assessing how changes in biotic interactions driven by warming-associated shifts in community composition are altering ecosystem functions in the Arctic. In particular, the changes that we observed in the overall structure of the arthropod community (namely the increase in herbivores and reduction in detritivores) suggest that climate-related effects on high-arctic arthropods could ultimately affect ecosystem processes such as decomposition, nutrient cycling and primary productivity [67]. Given that the Arctic is a reservoir for approximately half of the global pool of soil organic carbon [130–132], the patterns we document here could have important consequences not only for regional but also for global carbon dynamics.

## Supplementary Material

Imputation of average summer temperature at Zackenberg in 1995

## Supplementary Material

Changes in raw climate variables over study period at Zackenberg, Greenland

## Supplementary Material

Variation in arthropod abundances at Zackenberg

## Data accessibility

All data used here, including the arthropod community composition data and the climate (temperature) data are publically available at http://data.g-e-m.dk/.
