# Supplementary material for: Differential arthropod responses to warming are altering the structure of Arctic communities
Source: R Soc Open Sci. 2018 Apr 18;5(4):171503. doi: 10.1098/rsos.171503 (PMC5936898; doi:10.1098/rsos.171503)
Supplement: Imputation of average summer temperature at Zackenberg in 1995 [file rsos171503supp1.docx]

Amanda M. Koltz, Niels M. Schmidt, and Toke T. Høye

Differential arthropod responses to warming are altering the structure of arctic communities

Royal Society Open Science

**Electronic supplementary material 1:**

**Imputation of average summer temperature in 1995**

Summer temperature data from the year prior to the start of our study (1995) were unavailable from the Zackenberg climate station. However, gridded temperature data (0.5° resolution) for the Zackenberg area were available during the same period from the Climate Research Unit (CRU) TS3.23 Dataset (downloaded from rudata.uea.ac.uk/cru/data/hrg/cru_ts_3.23/, accessed Feb. 25, 2016). In order to include this measure in our models, we imputed the average summer temperature from 1995 based on the correlation between these two data sets (Fig. S1).


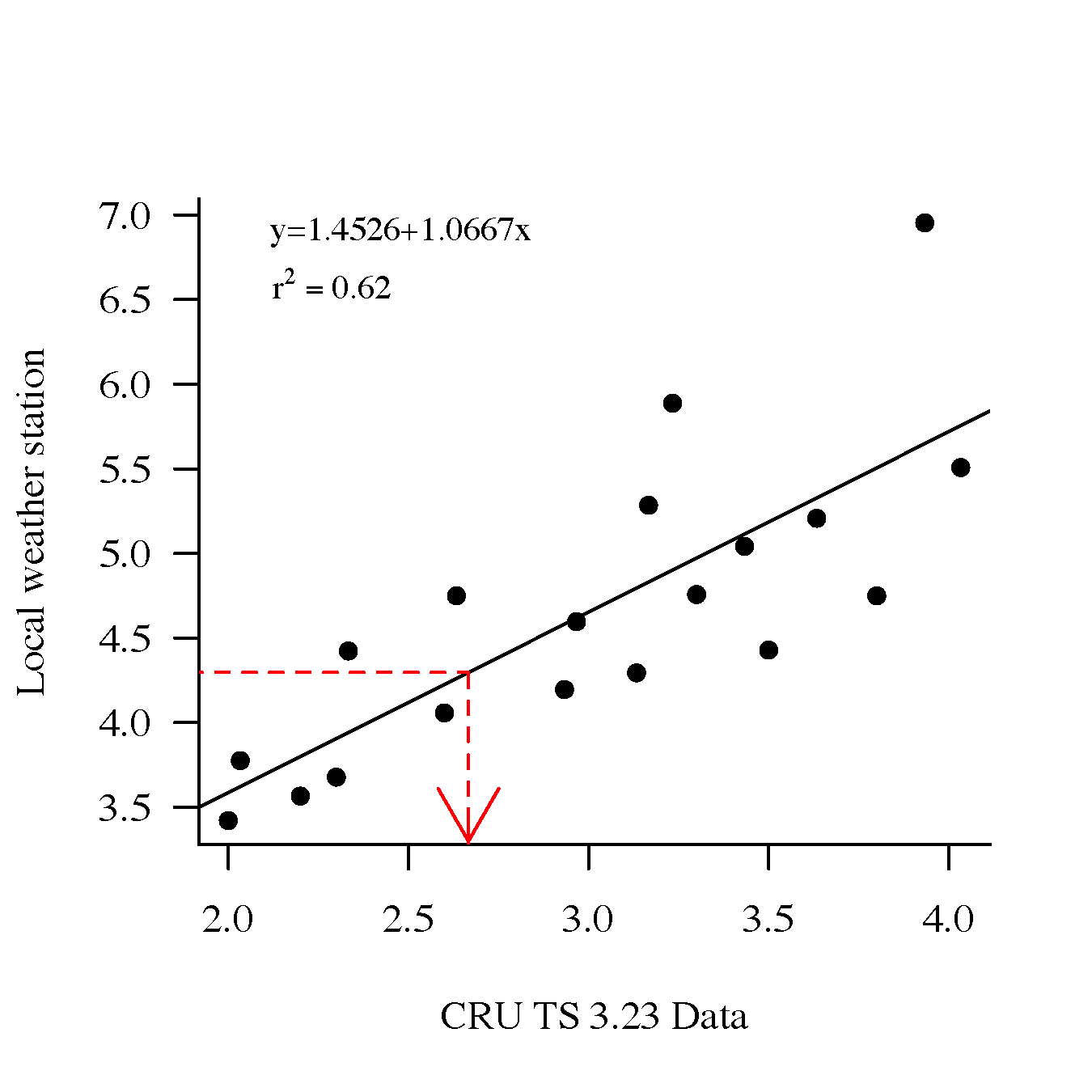


**Figure S1.** Imputation of the average local summer temperature at Zackenberg for year 1995. Zackenberg’s local weather station temperatures for the years 1996 to 2014 were regressed against the temperatures for Zackenberg in the global 0.5 degree by 0.5 degree data set from the University of East Anglia's Climate Research Unit (CRU TS v. 3.23). As depicted in red, the local temperature for 1995 was then estimated as the expected local weather station value given the observed CRU value.
