# Supplementary material for: Differential arthropod responses to warming are altering the structure of Arctic communities
Source: R Soc Open Sci. 2018 Apr 18;5(4):171503. doi: 10.1098/rsos.171503 (PMC5936898; doi:10.1098/rsos.171503)
Supplement: Changes in raw climate variables over study period at Zackenberg, Greenland [file rsos171503supp2.docx]

Amanda M. Koltz, Niels M. Schmidt, and Toke T. Høye

Differential arthropod responses to warming are altering the structure of arctic communities

Royal Society Open Science

**Electronic supplementary material 2:**

**Changes in raw climate variables over study period at Zackenberg, Greenland**

**Table S2.** Linear regression output for the climate variables that were incorporated into the PCA (average seasonal temperatures, winter duration, and the number of winter-freeze thaw events; see main text) from Zackenberg, Greenland over the study period of 1996-2014.

| **Response** | **Coeff.** | **SE** | **df** | **t** | ***p*-value** | **Multiple r^2^** |
| --- | --- | --- | --- | --- | --- | --- |
|  |  |  |  |  |  |  |
| **Fall** |  |  |  |  |  |  |
| Intercept | -193.505 | 98.626 | 16 | -1.96 | 0.067 | 0.185 |
| Year | 0.094 | 0.049 | 16 | 1.91 | 0.075 |  |
|  |  |  |  |  |  |  |
| **Winter** |  |  |  |  |  |  |
| Intercept | -177.205 | 93.197 | 16 | -1.90 | 0.075 | 0.153 |
| Year | 0.079 | 0.046 | 16 | 1.70 | 0.108 |  |
|  |  |  |  |  |  |  |
| **Spring** |  |  |  |  |  |  |
| Intercept | 35.656 | 163.405 | 16 | 0.22 | 0.830 | 0.005 |
| Year | -0.022 | 0.082 | 16 | -0.275 | 0.787 |  |
|  |  |  |  |  |  |  |
| **Summer** |  |  |  |  |  |  |
| Intercept | -197.380 | 60.390 | 16 | -3.268 | 0.005 | 0.412 |
| Year | 0.101 | 0.030 | 16 | 3.346 | 0.004 |  |
|  |  |  |  |  |  |  |
| **Winter duration** | |  |  |  |  |  |
| Intercept | -816.704 | 1525.521 | 16 | -0.54 | 0.600 | 0.027 |
| Year | 0.507 | 0.761 | 16 | 0.67 | 0.515 |  |
|  |  |  |  |  |  |  |
| **Winter freeze-thaw events** | | |  |  |  |  |
| Intercept | 1124.462 | 567.710 | 16 | 1.981 | 0.065 | 0.194 |
| Year | -0.556 | 0.283 | 16 | -1.965 | 0.067 |  |


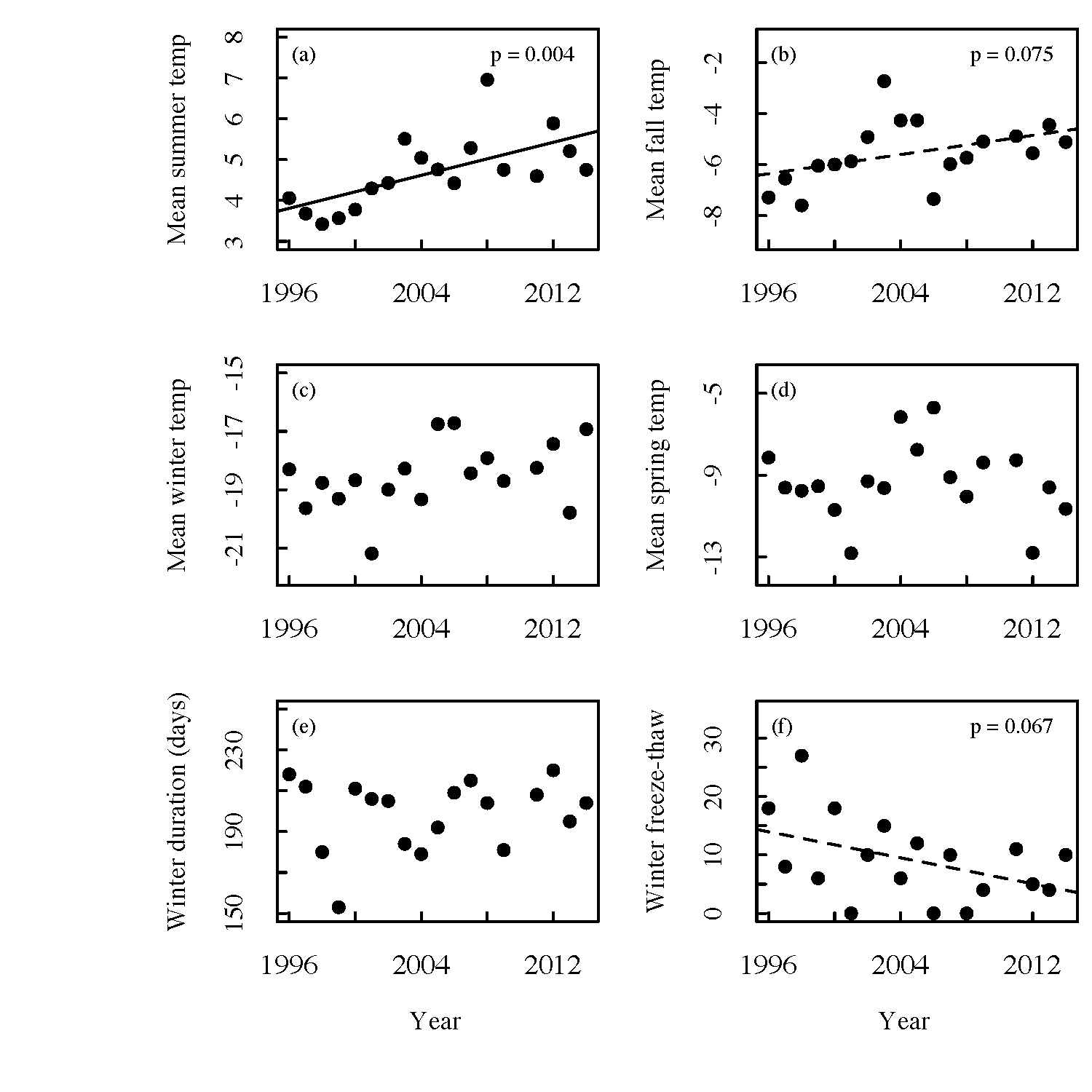


**Figure S2.** Variation in average temperatures during (a) summer, (b) fall, (c) winter, and (d) spring, as well as annual variation in (e) winter duration and (f) number of winter-freeze thaw events at Zackenberg, Greenland during the study period of 1996-2014. The solid regression line for summer temperature denotes a significant change over time; dotted lines for fall and the winter freeze-thaw events denote the marginally significant changes in these variables over the study period. See main text for detailed description of variables and Table S2 for linear regressions output of climate variables over the study period.
