# Supplementary material for: Differential arthropod responses to warming are altering the structure of Arctic communities
Source: R Soc Open Sci. 2018 Apr 18;5(4):171503. doi: 10.1098/rsos.171503 (PMC5936898; doi:10.1098/rsos.171503)
Supplement: Variation in arthropod abundances at Zackenberg [file rsos171503supp3.docx]

Amanda M. Koltz, Niels M. Schmidt, and Toke T. Høye

Differential arthropod responses to warming are altering the structure of arctic communities

Royal Society Open Science

**Electronic supplementary material 3:**

**Variation in arthropod abundances at Zackenberg (1996-2014)**

In all analyses, our measure of arthropod abundance was the total number of animals per taxonomic group that was caught between June-August of each sampling year (1996-2014). Capture numbers within plots were standardized across sampling years by transforming total annual specimen counts to individuals per trap per day. Here we show mean animal abundance/day for each of these taxonomic groups by habitat type to highlight the large number of small-bodied animals (namely Collembola and Acari) that were caught in our pitfall traps (Fig. S3).


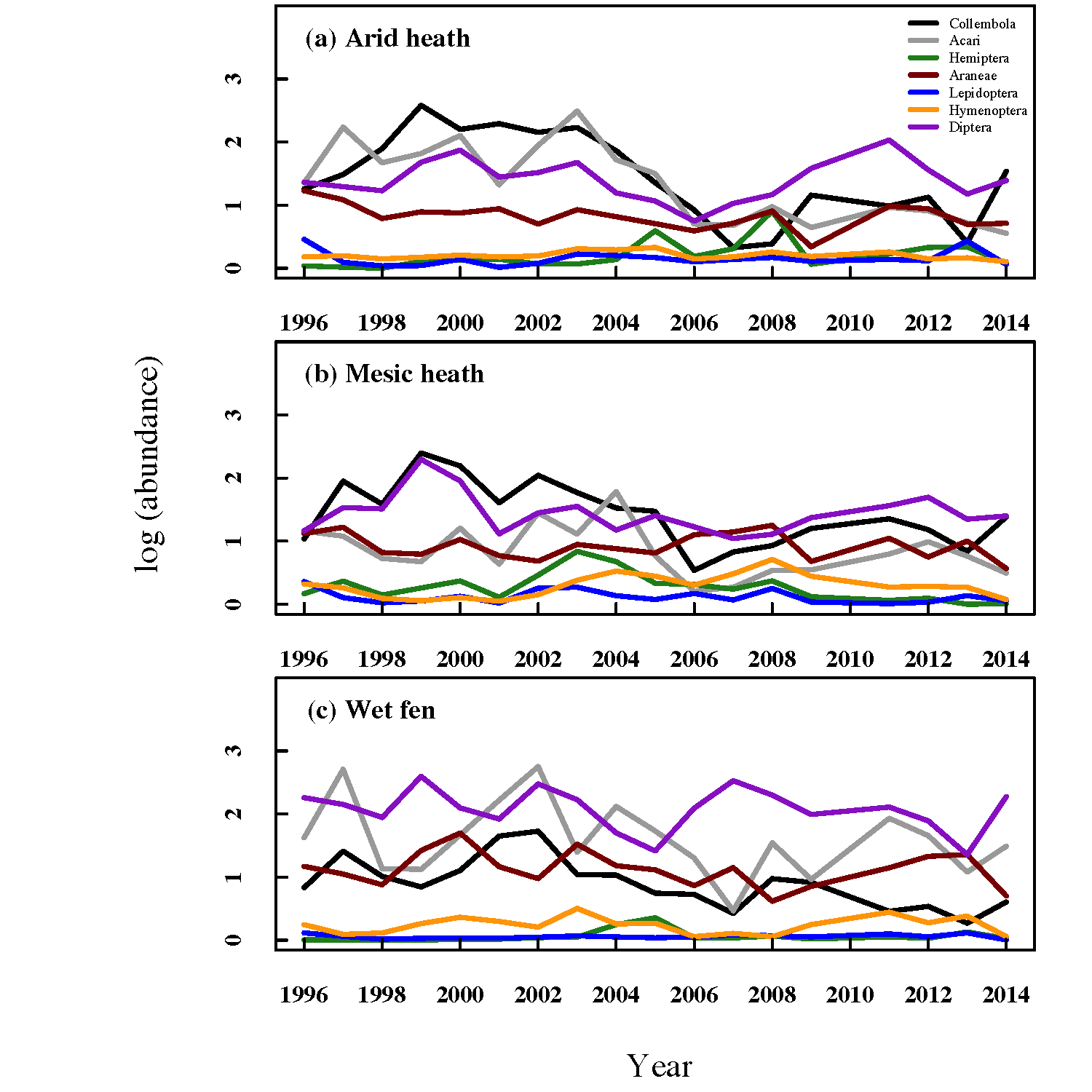


**Figure S3.** Average number of arthropods caught per day over the study period of 1996-2014 in the (a) arid heath, (b) mesic heath, and (c) wet fen habitats in Zackenberg. Animals were sampled using pitfall traps from June-August of each year. Capture numbers represent the total number of arthropods caught over the whole season, corrected by the number of trapping days within a given year.
